# Understanding Deformation Behavior in Uniaxial Tensile Tests of Steel Specimens at Varying Strain Rates

**DOI:** 10.6028/jres.126.050

**Published:** 2022-02-22

**Authors:** Dilip K. Banerjee, Mark A. Iadicola, Adam Creuziger

**Affiliations:** 1 National Institute of Standards and Technology, Gaithersburg, MD 20899, USA

**Keywords:** deformation, finite element analysis, Johnson-Cook model, localization, optimization, strain, strain rate, temperature, tensile test

## Abstract

Uniaxial tensile tests are routinely conducted to obtain stress-strain data for forming applications. It is important to understand the deformation behavior of test specimens at plastic strains, temperatures, and strain rates typically encountered in metal forming processes. In this study, the Johnson-Cook (J-C) flow stress model was used to describe the constitutive behavior of ASTM International (ASTM) A 1008 steel specimens during uniaxial tensile tests at three different average strain rates (10ˉ⁵ sˉ¹, 10ˉ³ sˉ¹, and 10ˉ¹ sˉ¹). The digital image correlation (DIC) technique was used for displacement and strain measurement, and two-dimensional (2D) infrared (IR) imaging was employed for temperature measurement. Separate optimization studies involving relevant finite element (FE) modeling with appropriate measured data yielded optimum values of convective heat transfer coefficients, J-C parameters, and inelastic heat fraction variables. FE modeling employing these optimum parameter values was then used to study the mechanical behavior. While FE predictions matched measured strain localization and thermal field very well in the intermediate- and low-rate experiments, the high-rate test showed narrower strain localization and a sharper temperature peak in the experiment. Possible use of a higher steel thermal conductivity value and/or exclusion of material inhomogeneities may have resulted in discrepancies between computed and measured temperature and strain fields. The study shows that an optimized set of parameters obtained with a controlled test could be reasonably applied for other tests conducted at very different strain rates.

## 1 Introduction

Sheet metal forming is a very common metal processing operation in which a high degree of precision is desired relative to the geometry and mechanical properties of the final product. In order to reduce time and costs associated with traditional trial-and-error methods (*e.g.*, die tryouts), modeling of material behavior during forming is increasingly being conducted with the finite element (FE) method. Therefore, FE models need to include accurate constitutive laws and the capability to predict the formability limits of the material if they are to be used in production mode. Additionally, automotive companies are actively interested in the increased use of advanced lightweight materials such as advanced high-strength steels (AHSS), aluminum alloys, *etc*., as sheet metal components. However, accurate material models are needed before these materials can be widely used. These constitutive data need to be obtained at strains, strain rates, and temperatures corresponding to those used in the forming processes.

Although sheet metal forming rarely involves uniaxial stretching, uniaxial tensile tests are often useful in understanding behavior of sheet metals. Uniaxial tensile testing still remains the most widely used method for understanding the mechanical behavior of materials and is routinely conducted to obtain stress-strain data for forming applications. The stress-strain constitutive law data needed for most forming operations often extend beyond the strain corresponding to the ultimate stress. These constitutive material data are regularly used in FE models constructed to study the mechanical behavior of specimens undergoing complex forming operations. However, the applicability of uniaxial stress-strain data for these complex scenarios is limited due to the fact that the effects of strain rates (*i.e.*, strain rate hardening/softening and plastic heating) are not often explicitly included in such constitutive models. A study of the influence of strain rates on strain localization and evolution of heat as a result of plastic work can provide insight into material constitutive behavior during real and complex forming operations [1–3]. This is because the flow stress of a material depends on both strain rate and temperature. The rise of temperature as a result of plastic work can aid in softening the material, especially during high-strain-rate plastic deformation.

Stress-strain data (constitutive relation) in uniaxial tensile tests are typically recorded up to the maximum force point (*e.g.*, point corresponding to the ultimate tensile strength [UTS]). The constitutive behavior beyond the UTS is assumed in the models typically used in FE programs. Since forming operations need stress-strain data beyond UTS, as mentioned earlier, the digital image correlation (DIC) technique is being used to provide strain data beyond that corresponding to the UTS. Current research efforts are focused on using DIC measurement data to fit constitutive models beyond the UTS and all the way to the failure point. Additionally, many factors are often ignored in conventional uniaxial tensile tests. Such factors should be included to enhance the accuracy of the constitutive models. As discussed previously, strain rate is one of these factors and is the focus of this study.

Thus, the aim of this study was to conduct uniaxial tensile tests of ASTM International (ASTM) A1008 steel specimens and model the material behavior using the Johnson-Cook (J-C) model [4] in commercial FE software, Abaqus^1^1 Certain commercial equipment, instruments, software or materials are identified in this paper in order to specify the experimental procedure adequately. Such identification is not intended to imply recommendation or endorsement by the National Institute of Standards and Technology, nor is it intended to imply that the materials, software or equipment identified are necessarily the best available for the purpose. [5]. The J-C model can include the effect of strain rate on the constitutive behavior of materials. The J-C model is calibrated by comparing the displacement, plastic strain, and temperature field in the specimen from one uniaxial experiment at one strain rate with those obtained by FE simulation. That calibrated model is then verified against other experimental results for the same material. Through this process, insight into the material behavior during localization is obtained. The main goal for the experimental and numerical simulation of uniaxial tensile tests at varying strain rates was to understand strain, temperature, and deformation localization leading to failure. The study included an optimization study for determining the optimum set of J-C model parameters, the inelastic heat fraction parameter (which controls the fraction of plastic work that is converted into heat), and the convective heat transfer coefficient. This was done with the objective of minimizing the difference between measured and predicted quantities along the centerline of the specimen as discussed later in the text. This study was part of ongoing efforts to develop a better understanding of deformation and failure mechanisms in biaxial tensile testing of advanced lightweight alloys [1–3].

The text is structured as follows. Section 2 describes experimental procedures; Sec. 3 describes the FE model for heat transfer and mechanical behavior, including the rationale behind the use of the J-C constitutive model, and it also explains the optimization approach used; Sec. 4 provides a comparison between measured and computed results; and, finally, Sec. 5 provides a summary.

## 2 Experimental Procedure

### 2.1 Loading

Uniaxial test specimens were made of cold-rolled ASTM A1008 steel having a thickness of 2.94 mm. Tensile tests were conducted in a cruciform (biaxial) testing machine [6]. In this machine, the load is applied using hydraulic actuators, which are controlled in orthogonal pairs [6]. Each of these actuators has 500 kN load capacity and has a ±50 mm displacement range from a reference distance of 640 mm between grip faces on the *X* or *Y* axis. Details of loading and *in situ* data acquisition using the DIC system are described in Ref. [6]. In the present study, tests were conducted using displacement control.

**Fig. 1:**
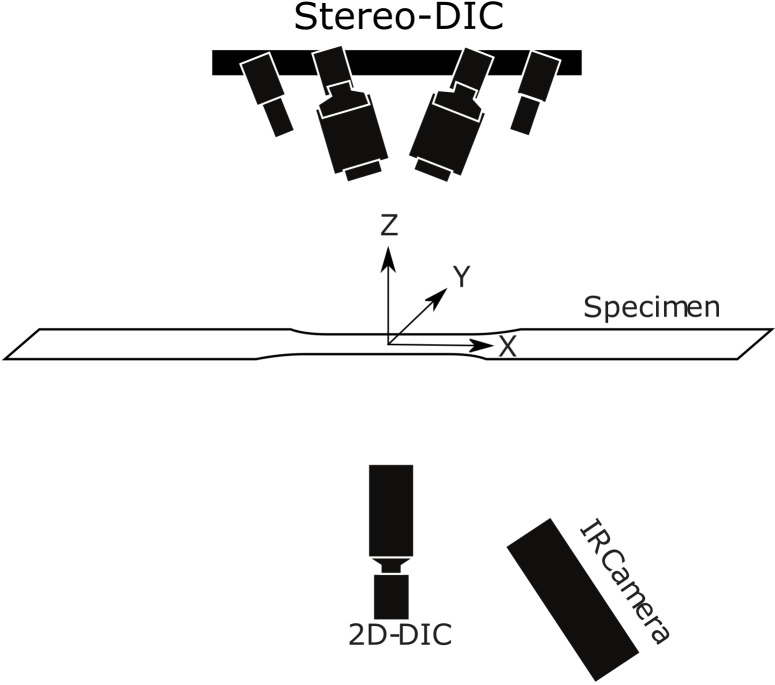
Schematic showing experimental setup, including a uniaxial tensile specimen along with DIC cameras and IR camera system.

Uniaxial tensile tests were performed in the rolling direction of the sheet. These tests were performed using the *X* axis of the cruciform machine and the DIC system (Fig. 1). The gauge section as designed has a nominally parallel length of 127 mm and width of 47.62 mm with radii of 63.5 mm to the 71.44 mm wide end tabs. The as-machined (by waterjet) widths and thicknesses were measured and used to calculate the initial cross-sectional area for each specimen.

**Fig. 2:**
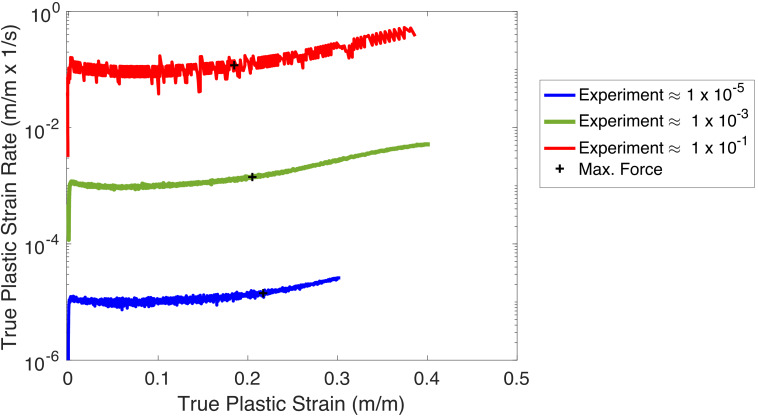
Strain rate *vs*. plastic strain for the three tests.

Uniaxial tensile tests were conducted using displacement control at three different nominal strain rates, hereinafter called high rate (with average strain rate of 10^−1^ s^−1^), intermediate rate (with average strain rate of 10^−3^ s^−1^), and low rate (with average strain rate of 10^−5^ s^−1^). The plots of instantaneous strain rate versus plastic strain for the three tests are shown in Fig. 2. These strains and strain rates are based on the average longitudinal strain over approximately 12 mm in the center of the parallel length. Note that the low-rate test did not go to failure, while the intermediate- and high-rate tests did reach failure.

### 2.2 Digital Image Correlation (DIC) Displacement and Strain Measurement

The three-dimensional (3D) displacement of the top surface of the specimen was measured with two stereo-DIC systems. Each of these systems has two cameras. One system is for a wide field of view out to the grips and the other is for a close-up field of view of approximately 70 mm by 65 mm. The systems measure the surface *U*, *V*, and *W* displacements in the *X*, *Y*, and *Z* directions, respectively (see Fig. 1). Note that DIC requires a portion of the surface on which to conduct correlations, and so the measurement points begin about 0.3 mm from the edges of the surfaces. Surface strains are calculated from the measured displacement fields. Note the following uncertainties in measurements: force ±0.025 kN; *U*, *V*, and *W* displacements: (1.5, 1.5, and 2.5) µm, respectively.

**Fig. 3:**
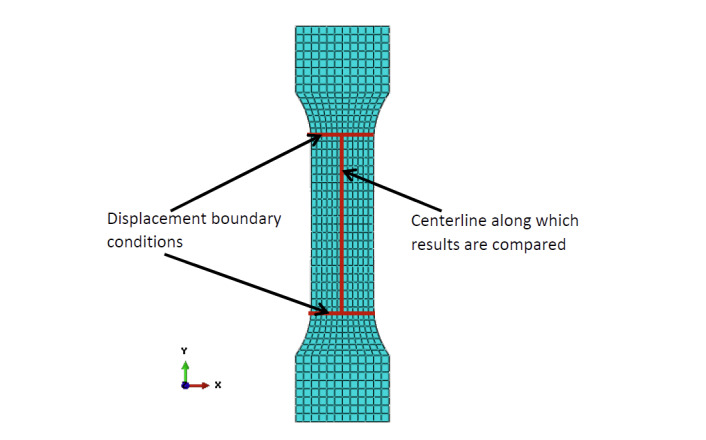
FE mesh of test specimen used for modeling three tensile tests, showing locations where displacement boundary conditions were used and where results were compared with test data.

Following each tensile test, history-dependent *U* values at locations approximately 63.5 mm away from the midpoint of the parallel length were extracted for subsequent use in finite element analysis (FEA) simulation (see Sec. 3.2). The displacement values were interpolated from the DIC field data at the FEA nodal coordinate values nearest to X = ±63.5 mm across the specimen width (see displacement boundary condition locations in Fig. 3). The use of DIC data as boundary conditions in FEA models is described in Ref. [7]. Additionally, axial displacements along the centerline of the parallel length at specific times into the test were extracted for comparison with FE results. The DIC measurement uncertainties in strain were: ±0.0005 m/m (in *xx*) to ±0.0007 m/m (*yy*). Stress uncertainty varied from ±0.45 MPa to ±0.8 MPa.

Although the stress state of the test is only uniaxial up to maximum force, it is desirable to estimate how the stress-strain behavior extends beyond this point. To that end, a true stress–true strain curve was created using the DIC results along the specimen width located at the point of necking and eventual failure. The true strain was based on the average strain along the width. The true stress was calculated by dividing the force by the current area at each point in time, where the current area was calculated using the strain distribution across the width and the assumption of volume conservation during plastic deformation. No correction was made for the multiaxial stress state or for the growth of voids, resulting in a slight overestimate or underestimate of stress, respectively. Such stress-strain data will be used in future FE simulations, and the results will be compared with those predicted by the J-C model used here. Note that the experimental stress-strain curves in this paper were computed using this approach.

### 2.3 Temperature Measurement

Noncontacting measurement of surface temperature can be performed using a two-dimensional (2D) infrared (IR) imaging system (FLIR model A655sc). For this reason, an infrared camera was added below the specimen in the cruciform system for use in monitoring adiabatic heating during plastic deformation or phase transformations (see Fig. 1). Temperature measurement data were extracted along the length of the specimen at specific times into the test for all three tests and following failure in the high-rate and intermediate-rate tests. Such data were used in FE models as explained later in Sec. 2.4 and Sec. 3.1. Note that the noise in temperature measurement with IR was: ± 0.1 °C (assuming an emissivity of 0.95). The emissivity was a result of the matte paint used on the underside of the specimen and was verified against a K-type thermocouple at room temperature and at one elevated temperature.

### 2.4 High-Rate Test Cooling Study

After the uniaxial tensile specimen failed during the high-rate test, the thermal profile of the specimen was continuously measured for a period of 9.6 s as it cooled. The goal was to determine the convective heat transfer coefficient that was to be used in the combined temperature-displacement FE modeling of the mechanical behavior in the uniaxial tensile tests as explained later in Sec. 3.2. The geometry of the deformed specimen following failure was used to develop the FE model of the specimen for modeling the heat transfer during cooling.

**Fig. 4:**
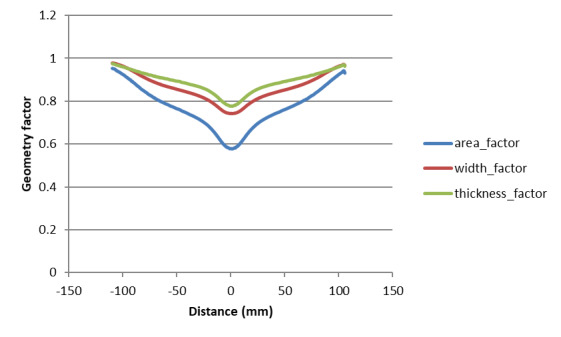
Variation of specific geometric factors (see Sec. 2.4 for explanation) along the length of the specimen in high-rate test just before failure based on DIC measurements and volume conservation.

When the sample failed during the tensile test, the geometry of the sample changed from the original one that existed at the beginning of the test. Variation of the measured width, thickness, and cross-sectional area (from the DIC measurements and assuming volume conservation) along the sample length at just before failure is shown in Fig. 4. The width factor is the ratio of the post-failure width at a location to the original width at that location. Similarly, the thickness factor is the ratio of the post-failure thickness at a location to the original thickness at that location, and the area factor is the ratio of cross-sectional area at a location to the original cross-sectional area at that location.

## 3 Finite Element (FE) Modeling and Optimization

**Fig. 5:**
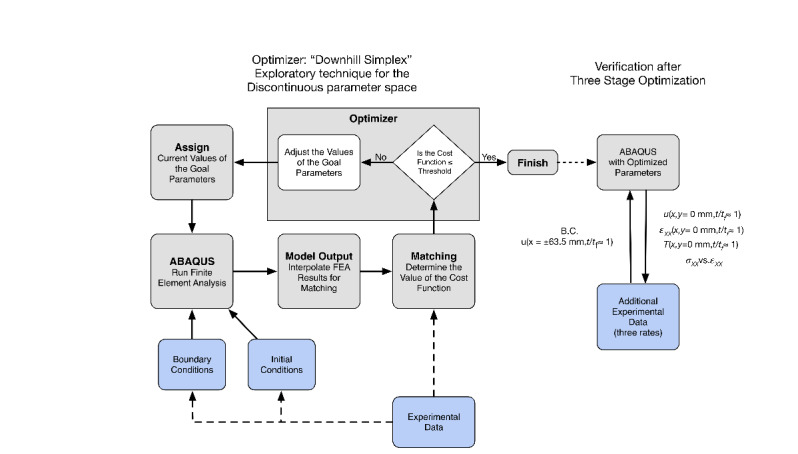
General optimization procedure (Note that “B.C.” stands for boundary conditions, *t* is time, *t_f_* is time to failure, *ε* is true strain, *σ* is true stress, symbols with subscript “xx” represent quantities in the axial or longitudinal direction).

A three-step optimization strategy was followed. Figure 5 is a schematic flow chart for each optimization step. The gray shaded boxes describe the optimization process, and the blue boxes show where portions of the experimental data are used. Initially, each of the three optimization stages begins with assigning initial values to the goal (design) parameters for that stage. Then, an FEA is performed using some amount of the experimental data for boundary and/or initial conditions. See Table 1 for the detailed steps used for each of the three stages of optimization. The output of the model is then sampled for specific data to compare with specific experimental data in the matching step. The quality of the match is judged through a cost (objective) function that the optimizer (Isight [8]) uses to either update the goal parameters or accept the values as acceptable within some threshold. Table 1 summarizes the three optimization stages, including the goal (design) parameters, fundamental changes in the model type, initial conditions, and the experimental data that were used as either input or validation of the results of the FEA.

### 3.1 Determination of Optimum Interfacial Heat Transfer Coefficient of Post-failure High-Rate Test Specimen

**Fig. 6:**
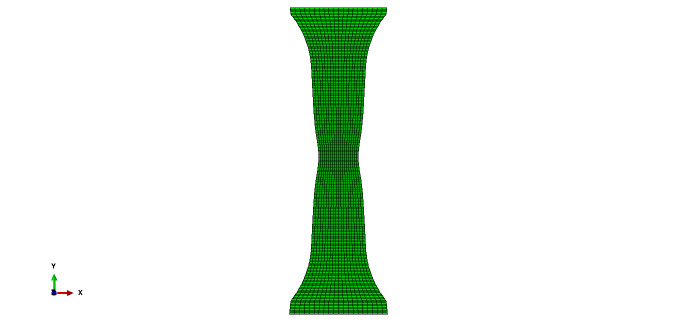
FE model of the shape of the test specimen just before failure used for post-failure thermal analysis.

This analysis used data from the high-rate test after specimen failure (when deformation had ceased, and the strain rate went to zero). An FE model of the specimen using the variation in final shape along the axial direction as shown in Fig. 4 was constructed in FE software [5]. This model was developed using Python scripting in FE software and is shown in Fig. 6. The model was constructed such that the volumes of the specimen in the experiment and model matched exactly. The varying widths of the specimen and cross-sectional areas along the length of the specimen were included appropriately in the model. For the thermal model, standard, reduced integration, linear hexahedral heat transfer elements (DC3DR) were used for FE discretization of the model. Standard constant thermophysical properties of ASTM A1008 steel were used in the model [9]. The temperatures along the sample length at failure (in 0.5 mm increments) from the high-rate test were extracted at the instant just before the failure point and used as initial conditions for the subsequent post-failure FE model thermal analysis. The FE model was built in half symmetry about the midplane in the thickness direction. The boundary conditions were applied as follows: (1) there was no heat flow through the symmetry plane and (2) the measured temperature at an axial location of *x* = ±107 mm was used as a temperature boundary condition (Dirichlet type) (see Stage 1 column in Table 1). All other exposed faces of the specimen were allowed to exchange heat with the surroundings using a Newtonian cooling type of heat-exchange equation. This interfacial convective heat transfer coefficient, *h_int_* (at sample/surrounding boundary), was the only unknown quantity at this analysis stage. Since a forced convection condition was applicable during the experiment, this *h_int_* parameter was allowed to vary between 10 and 50 W/m^2^/K [10]. At the point of failure in the high-rate test, the specimen was at an elevated temperature (with a peak near 160 °C), due to adiabatic self-heating, but the specimen was still under the same thermal ambient conditions. This allowed the model to be simplified to only a thermal heat transfer problem with measured boundary conditions and the initial condition. Therefore, the stage 1 problem used the DIC-determined specimen shape just prior to failure with an initial axial temperature distribution, *T*(*x*, *y*_avg_), based on the IR temperature field at failure. The matching step compared the temperature along the longitudinal axis in the model to the IR data at a time 9.6 s after failure, when substantial cooling had occurred. The optimized value of *h_int_* was found to be 25 W/m^2^/K. This optimized *h_int_* value was subsequently used in the FE mechanical simulation of tensile tests (high-, intermediate-, and low-rate tests) for verification as explained in Sec. 4.2.

**Table 1:**
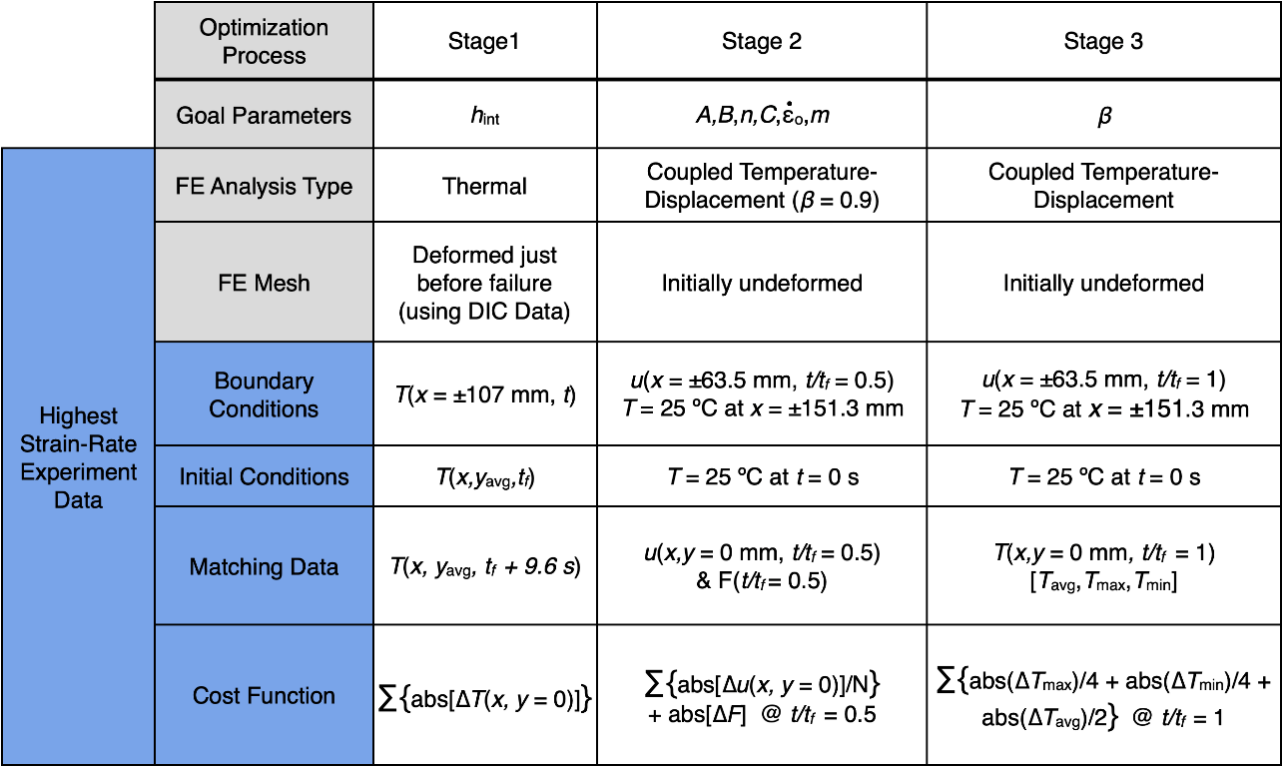
Summary of the three stages of optimization to determine the goal (design) parameters (Note that Δ represents the difference between measured and computed values of parameters).

### 3.2 FE Modeling of Deformation in Tensile Tests during Optimization (Stages 2 and 3)

The FE simulations of tensile tests during stage 2 and stage 3 optimization (Table 1) were conducted using dynamic, explicit formulation employing coupled temperature-displacement linear, hexahedral elements in FE software [5]. Both standard (C3D8T) and reduced integration elements (C3D8RT) were used. Two different models were constructed: one with four-element discretization along the thickness direction using C3D8T elements, and the other with two-element discretization along the thickness direction using C3D8RT elements (for both models in half symmetry). Two-element discretization in the thickness direction was used in order to improve computational efficiency. Commonly used steel elastic properties were assumed, including a Young’s modulus of 210 GPa and Poisson ratio of 0.3. The steel was modeled as an isotropic solid with viscoplastic behavior governed by the J-C [4] constitutive form as shown below:

$\sigma =\left(A+B\varepsilon^{n}\right)\left(1+C\;ln\left(\frac{\dot{\varepsilon }}{{\dot{\varepsilon }}_{0}}\right)\right)\left(1-{\left(\frac{T-T_{r}}{T_{m}-T_{r}}\right)}^{m}\right)\;$(1)

where σ is the flow stress, ε is the equivalent plastic strain, $\dot{\varepsilon }$ is the equivalent plastic strain rate, and ${\dot{\varepsilon }}_{0}$ is the reference strain rate measured at or below the reference temperature. The parameters *A*, *B*, and *n* describe the yield and plastic hardening behavior, while *C* describes the strain rate effects, and *m* controls thermal softening. $T_{r}$ is the reference temperature at which initial yield stress, *A*, is measured, and $T_{m}$ is the melting point. This particular model was chosen because it captures both the viscoplastic and thermal softening behavior using the least possible number of empirical parameters. The J-C constitutive model was originally developed empirically to describe the strain rate and temperature sensitivity of metals. The J-C equation is a highly useful constitutive model where the stress is given as an analytical function of strain, strain rate, and temperature. Due to its simplicity and relatively few material constants, many modifications of the J-C model have been proposed [11–12]. Inelastic heat fraction, *β*, is another input parameter that is used to determine how much plastic work is converted to heat, which is often not known precisely. Equation (2) provides the expression used to determine the temperature rise (∆T) during plastic deformation [13]:

$∆T=\;\frac{\beta }{\rho C_{P}}\int_{0}^{\varepsilon_{f}}\sigma \;d\varepsilon \;$ (2)

where ρ is the density, $C_{P}$ is the gravimetric specific heat, and $\varepsilon_{f}$ is the plastic strain corresponding to the failure point. The main consideration when using this J-C model is that it is essentially phenomenological. It attributes a power-law hardening behavior to the material and scales this description up or down, depending on the strain rate and temperature. It is not applicable for materials where the effects of strain rate and temperature on the flow stress are dependent on strain (*e.g.*, Al-5083, oxygen-free high-thermal-conductivity [OFHC] copper) [14–15]. The J-C constitutive model uses a particular type of isotropic hardening where the flow stress is assumed to have a multiplicative relationship with strain hardening, strain rate sensitivity, and thermal softening. A von Mises yield surface (with isotropic yielding) with associated flow is used in the implementation of the J-C model in FE software. Anisotropy of the material is not included in the model.

As mentioned in Sec. 3.1, an FE model was developed to take advantage of the mirror symmetry in the thickness direction. The model was developed in such a way that a regular mapped discretization was possible with hexahedral elements. Note that C3D8RT elements have one Gauss (integration) point per element located at the centroid of the element. Mesh seeding of the parts was done properly to obtain a reasonably fine mesh. Figure 3 shows a typical FE mesh of the tensile test specimen used for modeling the mechanical behavior.

For the half-symmetry model, appropriate boundary conditions were applied to the symmetry plane. On either end of the model, nodes that are about 63.5 mm away from the central point in axial directions were assigned history-dependent displacement boundary conditions obtained from DIC measurements during the tensile tests (see Sec. 2.2; Fig. 3). Note that all nodes along the depth direction were assigned the same history-dependent displacement boundary conditions as those on the surface. A fixed temperature of 25 °C was assigned to all nodes that were located at the axial ends of the end tabs in either direction. A convective boundary condition using a value of 25 W/m^2^/K (as discussed in Sec. 3.1) for the *h_int_* parameter was applied to all surfaces that were exchanging heat with the surroundings, as explained in Sec. 3.1. Note that heat diffusion within the specimen occurs by thermal conduction, while heat exchange with surroundings was assumed to occur by thermal convection. The following thermophysical parameters were used for the ASTM A1008 steel: density = 7890 kg/m^3^, thermal conductivity = 43 W/m/K, and heat capacity = 431 J/kg/K [9].

Following the completion of simulation, a path was defined along the centerline of the specimen surface (as shown in Fig. 3) similar to that viewed by the DIC system. Computed strain, displacement, and temperature measurement data were mapped to 21 equidistant points along this line for comparison with experimental measurements. This mapping was done for the load point corresponding to 50% of the sample failure time for the high-rate and the intermediate-rate tests and 50% of the final time in the low-rate test. As mentioned earlier, failure did not occur in the low-rate test.

### 3.3 Optimization of J-C Parameters and Inelastic Heat Fraction Parameter

The goal of this study was to determine the optimum J-C parameters and *β* that could be used to model the flow behavior obtained in all three tests. The optimization strategy used here has been presented briefly in Ref. [16]. Since the high-rate test is the most difficult problem to model, it was chosen as the test through which the optimum J-C parameters would be obtained. Toward this end, optimization software Isight [8] was used in conjunction with FE software. The optimization strategy (stage 2 and stage 3) was as follows. In stage 2, optimum values of parameters *A*, *B*, *C*, *m*, $\dot{\varepsilon_{0}}$, and *n* in Eq. (1) were obtained by minimizing the objective function (see Table 1) while using an assumed value of inelastic heat fraction (*β*) equal to 0.9. This comparison was made at the time corresponding to 50% of the duration of the test (the reader is referred to the end of this section for a better explanation of the minimization method). During this stage, the goal/design parameters (*i.e.*, J-C parameters shown in Table 1) were optimized. The matching and cost functions during stage 2 used the sum of the absolute difference between measured and computed average *U* displacements along the length of the specimen centerline and the absolute difference between measured and computed forces (*F*) at a time halfway to failure (*t*/*t_f_* = 0.5 s/s). Stage 3 of the optimization process used the optimum J-C parameters from stage 2 and *h_int_* values from Sec. 3.1 to determine the optimum values of the inelastic heat fraction, β, that minimized the differences between experimental and computed values of a weighted sum of the minimum, maximum, and median temperatures along the centerline at *t*/*t_f_* = 1 s/s. This weighted function was initially set at 50% weightage on median temperature differences and 25% weightage on each temperature difference of maximum and minimum temperatures obtained in experiments and in FE simulations at 21 points along the centerline. For both stages (stage 2 and stage 3 in Table 1), DIC-measured displacements at the limit of the parallel length (reduced width) of the specimen were used as displacement boundary conditions, *T* = 25 °C was the thermal boundary condition at the grips (*x* = ±151.3 mm), and the initial temperature was set to room temperature. Since temperature is the major response variable for the determination of *β*, only temperature was used in the matching step for stage 3, but only the minimum, maximum, and median temperatures, *T_min_*, *T_max_*, and *T_avg_*, were used in the cost function throughout the loading history.

The flow chart of the overall optimization procedure is shown in Fig. 5. In stage 2, the experimental displacement data collected along the centerline (note that location of measurement points matched corresponding nodal coordinates in the FE model) were read. In stage 3, the experimentally measured minimum, maximum, and median temperatures along the centerline were read. Next, initial values of J-C parameters and/or inelastic heat fraction, *β*, were defined (Table 2). Following each FE simulation, the desired FE output data (*e.g.*, axial displacement or temperatures) were extracted. A Java program was written to compute the differences between experimental and computed quantities. These difference values were minimized in the optimization module as shown in Table 1.

For the optimization, the “Downhill Simplex” exploratory technique was chosen mainly because this technique is well suited for discontinuous design spaces. This method employs a geometrically intuitive algorithm and samples the space across a subregion that moves in the direction of the opposite face of the simplex toward better solution away from the worst point [8]. Incidentally, a simplex is defined as a body in *n* dimensions consisting of *n* + 1 vertices. For example, the simplex is a triangle in 2D. As the optimization proceeds, the simplex moves downward toward the location of the minimum through a series

**Table 2:**
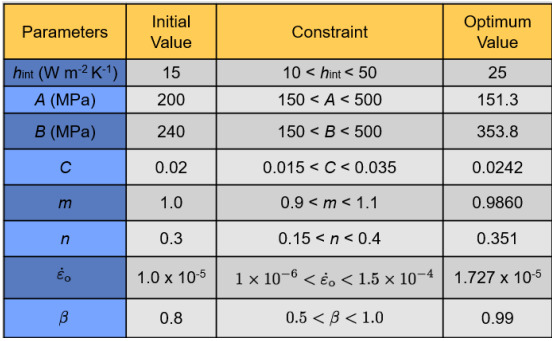
Values of initial and optimum J-C parameters and the inelastic heat fraction.

of steps. Further details of the technique are available in Ref. [8]. The current optimization procedure is classified as an inverse problem that has the following three components:

(1) uniaxial tensile test experimental data, which are the measured displacement/temperature data along the centerline and force data in the model;
(2) an FE model of the uniaxial tensile test, which provides the required output data; and
(3) an optimization procedure, which analyzes the discrepancy between experimental input data and simulation output data and minimizes this discrepancy.

With such an inverse analysis method, the coupling effect between strain rate and temperature can be analyzed. The temperature variation inside the specimen caused by plastic strain deformation is taken into consideration. The final fitting parameters (both J-C and β) are obtained when the cost function (*E*) describing the absolute difference between the predicted and the experimental values reaches the defined minimum value (see Table 1).

## 4 Verification Results and Discussion

In this section, verification results are discussed using plots of axial displacement, equivalent strain, and temperature profiles and axial true normal stress vs. axial true strain data. This is done by comparing plots of measured data with those obtained from FE simulations using parameter values (*i.e.*, *h_int_*, J-C parameters, and β) determined from the three stages of optimization (see Fig. 5), as shown in Table 2.

### 4.1 Thermal Cool Down after Fracture (High-Rate Test)

**Fig. 7:**
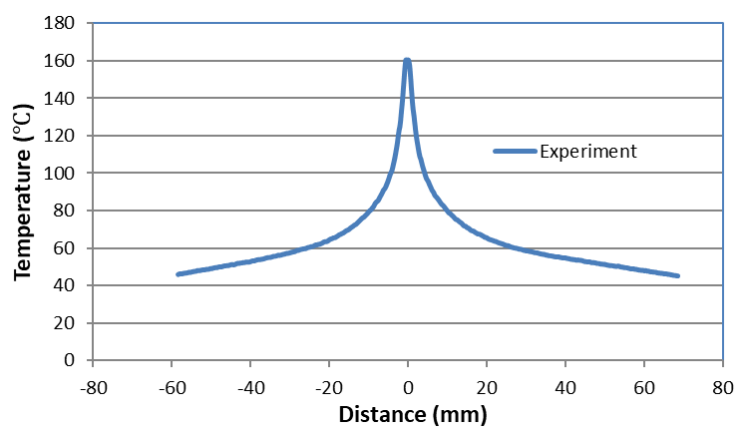
Measured temperature profile along the centerline just before failure in the high-rate test.

**Fig. 8:**
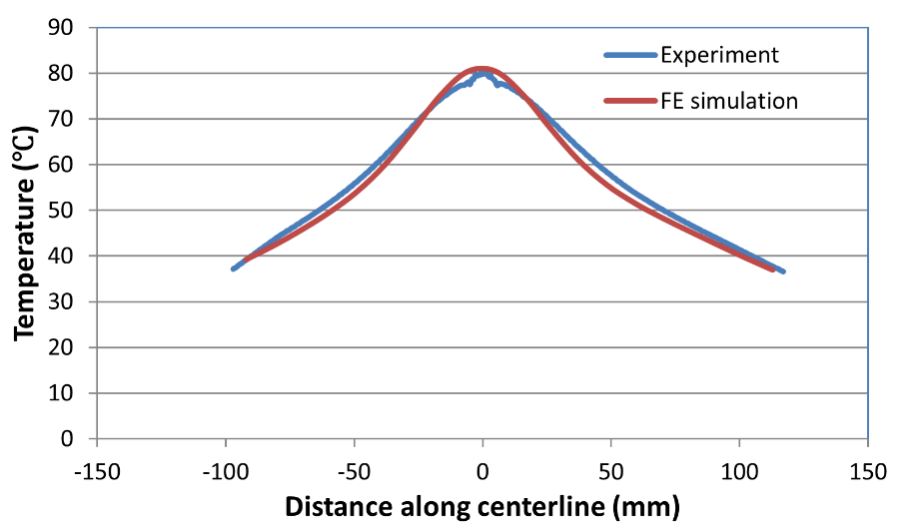
Measured and computed temperature profiles at the end of thermal test (about 9.6 s after mechanical failure) for the high-rate test.

In this section, thermal results for the high-rate test just prior to, and following, fracture are discussed. The initial temperature profile, shown in Fig. 7, was obtained by averaging the temperature measured across the width of the sample just before failure. It can be seen that most of the temperature is concentrated in the central region just before it failed. This temperature profile was used in the FE thermal model (Fig. 6) as an initial condition. Temperatures were considered to be uniform for each slice of the model that was 0.5 mm in length along the axial direction and through the entire depth. Following the procedure described in Sec. 3.1, a *h_int_* value of 25 W/m^2^/K was found to give the most optimum results (lowest variation between experimental and simulated temperatures). Figure 8 shows a comparison between the measured and computed temperature profiles along the centerline at the end of 9.6 s of the high-rate test (this is the final time when thermal data were recorded). Note the slight discrepancy between the computed and measured temperatures. This is possibly due to the slight error involved in the assignment of initial temperatures in the model, since, in reality, there is a temperature variation across the width of the specimen. However, the overall agreement seems to be reasonably good. Subsequently, this *h_int_* value was used in the coupled temperature-displacement FE simulation of the mechanical tests.

### 4.2 Mechanical Behavior Verification Results

Simulation of mechanical tests began with an initial guess for the J-C parameters. Meyers [13] provided appropriate values of these parameters for several practical alloys. Values for ASTM A1008 steel were not listed there, so reasonable values for other steels were used as initial guesses.

Table 2 lists the initial values, with ranges specified (constraints), and optimum values of the J-C parameters obtained following the procedure explained in Sec. 3.3 (stage 2 of the optimization). The constraint ranges were set based on values reported by Meyers [13]. Optimum values of the J-C parameters were then used in the subsequent exercise to optimize the values of the inelastic heat fraction parameter, β, as shown in Table 2 (stage 3 of optimization). Note that most of the energy associated with plastic deformation is typically converted into heat; only a small fraction is stored as residual internal work within the microstructure (*e.g.*, dislocations and vacancies). The value of 0.99 for β (as obtained in this study) is consistent with that typically obtained for high-strain-rate experiments [17]. These same, optimized values of J-C parameters and β were used for the intermediate-rate and the low-rate test simulations.

**Fig. 9:**
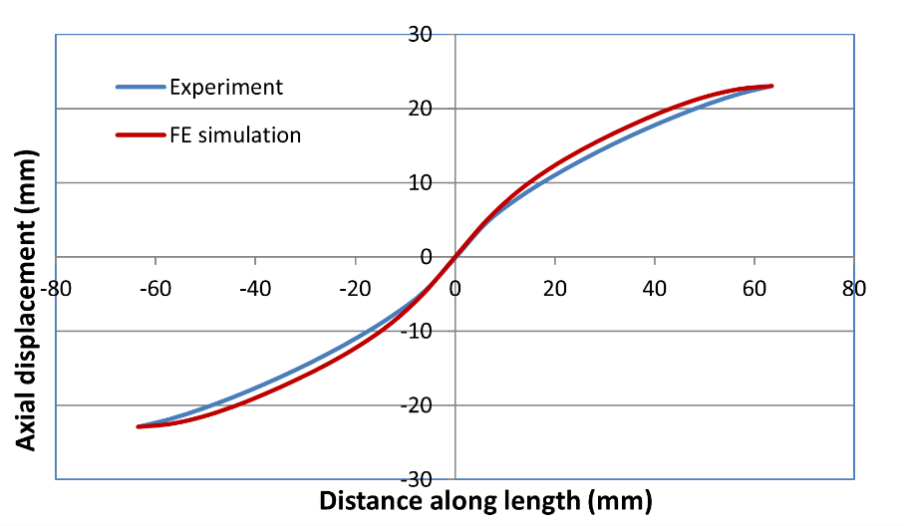
Axial displacement along centerline in the high-rate test just before failure.

**Fig. 10:**
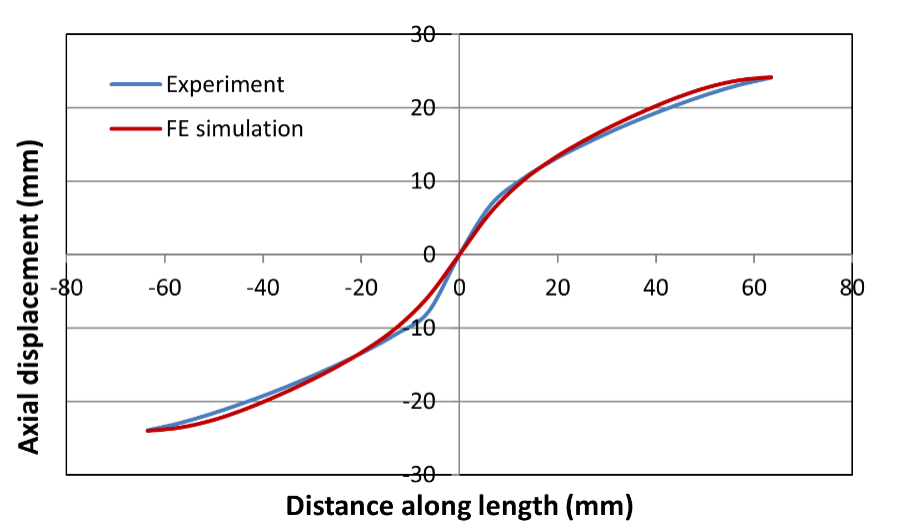
Axial displacement along centerline in the intermediate-rate test just before failure.

**Fig. 11:**
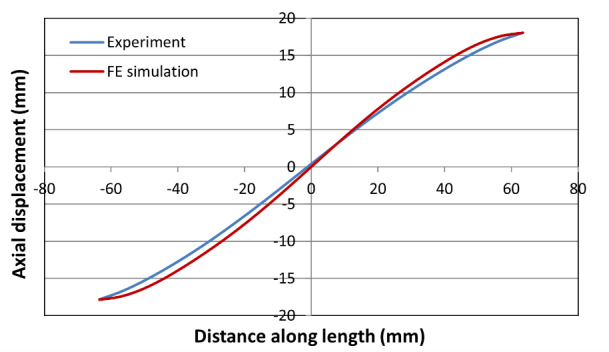
Axial displacement along centerline in the low-rate test at the end of the test.

Figures 9, 10, and 11 show plots of both computed and measured axial displacement profiles along the centerline for the high-rate, intermediate-rate, and the low-rate tests, respectively. For the high-rate and the intermediate-rate tests, they were taken at the point just before failure. For the low-rate test, they were taken at the end of the test (at 24,700 s into the test). These plots show good agreement between experimental and computed values. The match is especially good in the central neck region for the high-rate test because the model parameters were optimized for this high-rate test. However, the agreement in the central neck region is also fairly good for the intermediate-rate and low-rate tests. It appears that the neck region is slightly shifted away from the central point in the low-rate test. Note that no local imperfection was used in the model. The model appears to match the slope of the axial displacement profile in the neck regions that was obtained in these experiments.

**Fig. 12:**
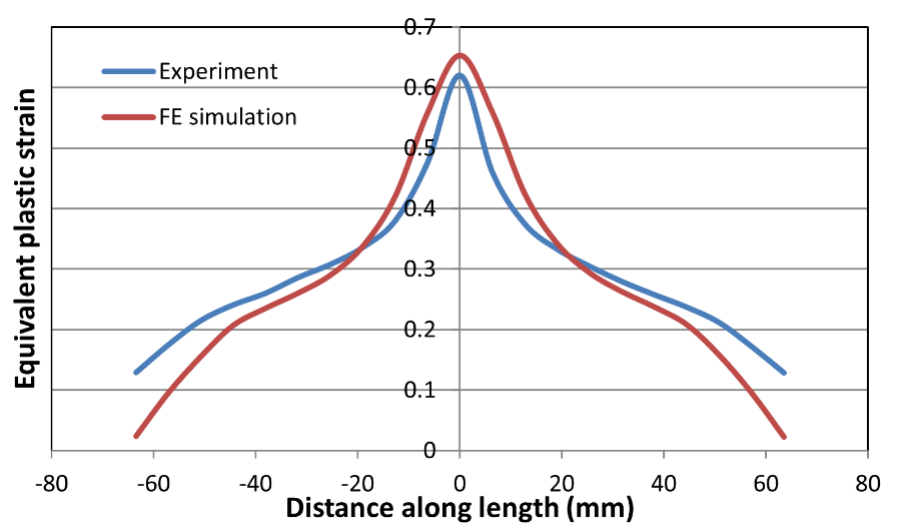
Equivalent plastic strain for the high-rate test just before failure.

**Fig. 13:**
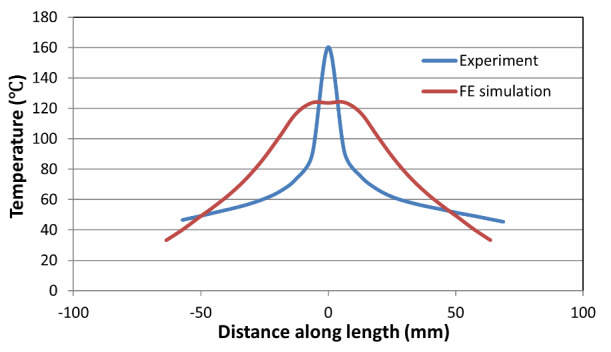
Thermal profile along the centerline in the high-rate test just before failure.

**Fig. 14:**
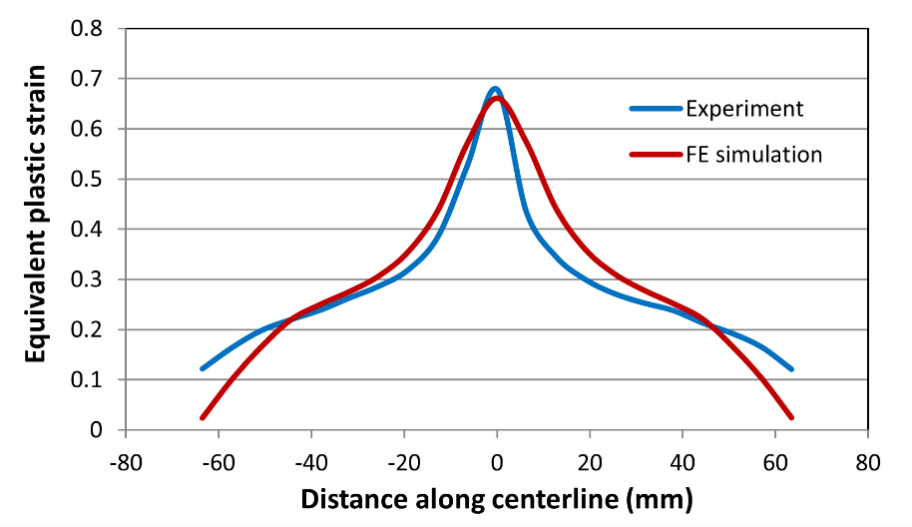
Equivalent plastic strain for the intermediate-rate test just before failure.

Figures 12 and 13 show the comparison of equivalent von Mises plastic strain and temperatures along the centerline for the high-rate test just before fracture occurred. Note that FE simulation provides equivalent plastic strain, while the experimental data include both elastic and plastic equivalent plastic strains (the contribution of the elastic component is very small). The peak value of equivalent strain is slightly higher in the FE model. However, the length of the overall displacement localization region is narrower in the FE model than it is in the experiment. The same is true for the intermediate-rate and low-rate test results, shown in Figs. 14 and 16. However, qualitatively speaking, there is a good match in the overall equivalent plastic strain between the measured and computed values, as seen by comparing the area under the curves. This is true for the high-, intermediate-, and low-rate tests (see Figs. 12, 14, and 16).

Figure 13 shows a comparison between the measured and computed temperatures along the centerline for the high-rate test. Note that the optimization was conducted to match a weighted sum of minimum, median, and maximum temperatures along the centerline. Note also that the IR camera system can record a maximum temperature of 160 °C. Thus, the actual temperature could have exceeded this temperature measurement limit. Although the average temperature rise in the model compares well with that in the experiment, the experiment shows a narrow band of localized plastic deformation in the neck that was not accurately captured by the J-C model used in this study. Since the model includes a coupled temperature-displacement analysis, heat transport through conduction and convection appears to have diffused the heat away from the neck region rather quickly. In particular, higher thermal conductivity values may have resulted in more rapid heat transport away from the central region than predicted. This possibility needs further investigation. Note that model predictions do not quite match experimental values at either end (as shown in Fig. 13) because the model uses far-field temperature boundary conditions at the axial ends of the end tabs.

**Fig. 15:**
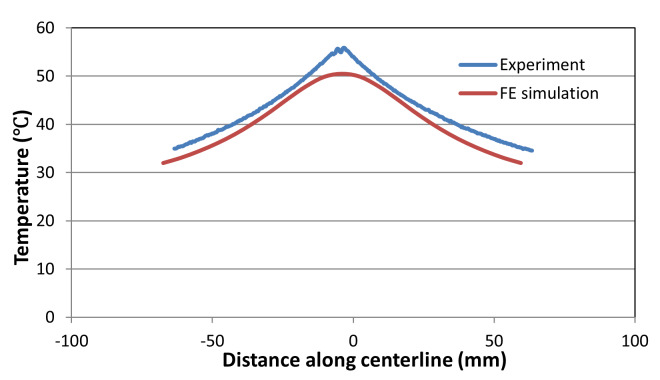
Thermal profile along the centerline in the intermediate-rate test just before failure.

**Fig. 16:**
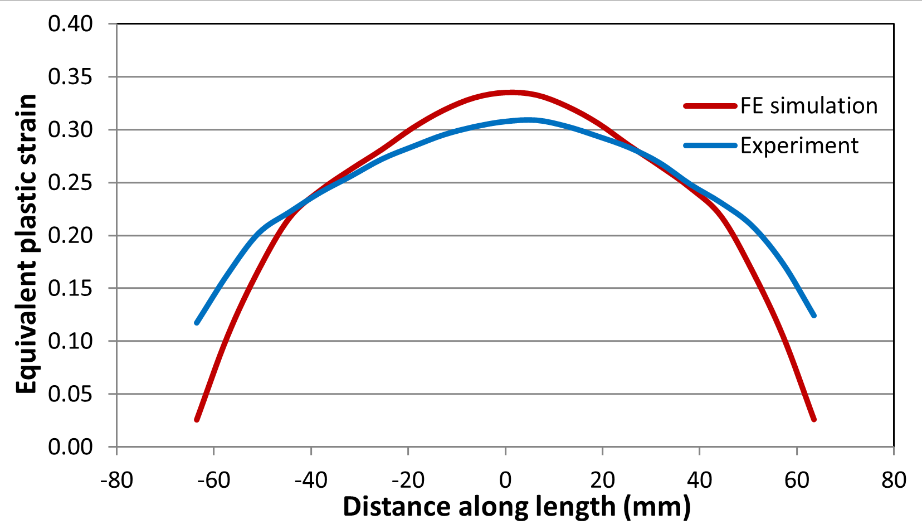
Equivalent plastic strain for the low-rate test at the end of the test.

**Fig. 17:**
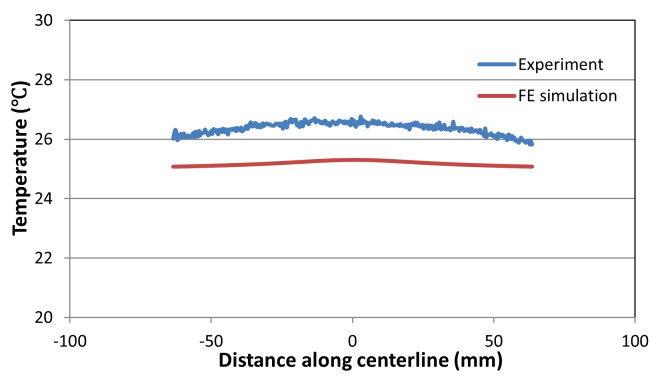
Thermal profile along the centerline in the low-rate test at the end of the test.

Figure 14 shows a very good match in the overall shape as well as peak values between measured and computed equivalent plastic strains for the intermediate-rate test. Note the slight disagreement in the location of the peak in the measured and computed curves. This discrepancy is about 4 mm in the axial direction. Figure 15 shows the measured and predicted thermal profile along the centerline for the intermediate-rate test at failure point, where computed points were displaced axially by 4 mm to coincide with the peak temperature locations. Figure 15 shows good agreement between the measured and predicted thermal profile, although the measured values are slightly higher consistently. Figure 16 shows a plot of comparison between the measured and computed equivalent plastic strain for the low-rate test. Again, there is a reasonably good agreement. A comparison between the measured and computed temperature fields is shown in Fig. 17. As expected, the temperature rise is minimal in this test because of long duration and very low strain rate. The actual small rise seen in the experiment was likely due to the heat from lighting over the long duration of the test. At these low rates, the stress-strain curve is not greatly affected by the rate of strain.

A disadvantage of tensile tests for the study of the behavior of materials at large strains (as in metal working processes) is that the strain rate is not constant (see Fig. 2) up to the fracture point due to the necking that occurs in ductile materials. The problems related to necking are further accentuated at high strain rates because adiabatic heating becomes localized in the necked region. In dynamic experiments, the temperature rise can be as high as 200 °C, or even higher for even higher strain rates. This temperature rise softens the material, leading to increased flow in the necked region. This effect thus limits the uniform strain that can be imposed. Such an effect complicates the study of the effect of strain rate on strain hardening and the eventual ductile fracture. Note that the localization is related principally to two factors: (1) material inhomogeneities (second phase particles, surface defects, *etc*.) and (2) constitutive behavior (material strain hardening, softening, and rate sensitivity). In this study, material inhomogeneities were not considered, which could possibly explain the discrepancy seen between measured data and the model prediction of localization.

**Fig. 18:**
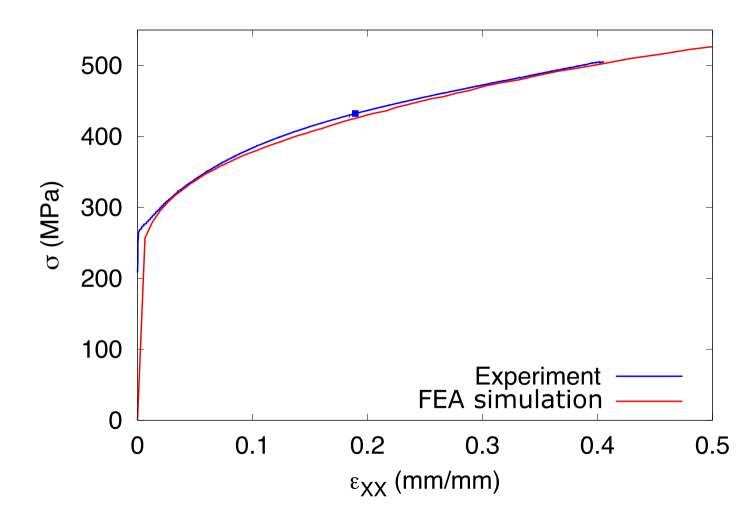
Comparison between measured and computed axial true stress *vs*. axial true strain values in the high-rate test using optimized values of all the parameters that were studied. The blue dot is the point of the maximum force.

Thermal softening can result in localization of flow in narrow bands. As Ref. [17] suggests, if one of these bands deforms more than another adjoining band, then the greater heating as a result of plastic deformation will lower the flow stress in this band, thereby cause even more concentration of flow and local heating in this band. This is probably the reason why there is a large difference in peak temperatures between measured and computed values in this study (Fig. 13). While FE simulation predicts a more gradual drop in equivalent strains and temperatures away from peak, the measured values show a very sharp drop on either side of the peak temperature location. This discrepancy might be better understood by using different constitutive models such as the mechanical threshold model (MTS [18]) and the Zerilli-Armstrong model [15]. These two models are well-known physics-based constitutive models, as opposed to the J-C model, which is empirical. In fact, Zerilli-Armstrong [14] compared the results of their model with that of the J-C model for predicting the radial strain profile (radial strain plotted against distance from the impact end) of an iron specimen (Taylor specimen [19]) impacted at 221 m/s. The Zerilli-Armstrong model provided a better correlation with experimental results for body-centered cubic (bcc) materials. Figure 18 shows a comparison of plots of axial stress *vs*. axial normal strain in the high-rate test specimen. The results show a good match (especially in hardening slopes and overall flow stress evolution) because the cost function in the optimization minimized the difference in forces between measured and computed forces in this test. However, the computed stresses for the intermediate-rate test and low-rate test are consistently higher than the measured stresses (not shown here). This is possibly due to the lower computed temperature rises in the intermediate-rate and the low-rate tests (see Fig. 15 and Fig. 17).

## 5 Summary

Uniaxial tensile tests of cold rolled ASTM A1008 steel were conducted at three different nominal strain rates: 10^−5^ s^−1^, 10^−3^ s^−1^, and 10^−1^ s^−1^. Time-varying displacement, strain, and temperature data were collected using DIC for displacement and strain measurements and an IR system for temperature measurements. Force *vs*. displacement data were also recorded during tests. An optimization procedure, in conjunction with heat transfer analysis of the post-failure temperature dissipation in the high-rate test, was conducted to obtain the optimum value of the convective heat transfer coefficient. This analysis used the sample geometry near failure. Subsequently, coupled temperature-displacement FE models of these test specimens were constructed employing the Johnson-Cook constitutive model for studying mechanical behavior. The FE model used measured history-dependent boundary conditions at either end of the central gauge section. In addition, fixed temperature boundary conditions were applied at either end of the specimen (toward the axial ends of the end tabs). Convective heat exchanges were allowed to occur during the modeling of mechanical behavior in the three tensile tests. The FE software was coupled with the optimization software to determine the optimum values of the J-C parameters and the inelastic heat fraction parameter. This was achieved by minimizing the difference between computed and measured values of displacement and temperatures, respectively, along the centerline of the sample and the difference between measured and computed forces in the high-rate test. These sets of optimum parameter values were subsequently used for the FE analysis of mechanical behavior in all three tests.

Computed displacement values along the centerline obtained with optimum parameter values agreed reasonably well with measured values in all three tests. While computed von Mises equivalent plastic strains in the intermediate-rate test agreed very well with measured values, a reasonably good match was seen in the low-rate test. Although the match in localization of strain in the high-rate test was not excellent, there was a good match in the overall trend. Both high-rate and intermediate-rate test results showed wider localization in the model predictions. Computed temperature profiles in the intermediate-rate and low-rate tests agreed well, although the measured values were slightly higher. In the high-rate test, the maximum measured temperature was substantially higher than that obtained with the FE analysis. However, the average computed temperature rise along the centerline agreed well with the experiment. The discrepancy in the temperature profile in the high-rate test may be attributed to the fact that material inhomogeneities were not included in the model and/or incorrect steel thermal conductivity values were used, which could play an important role in strain localization. A less accurate thermal boundary condition at the grips may also have contributed to this discrepancy. This study also shows that the optimized set of J-C parameters obtained from a single high-rate test produced reasonable results for the intermediate- and low-rate tests. In the future, the MTS model [16] and/or the Zerilli-Armstrong [15] model will be used to improve the accuracy of model predictions. A comparison of measured and computed axial stress *vs*. axial strain for the high-rate test agreed well, while the computed stresses for the intermediate-rate and low-rate tests were consistently higher than measured stresses, possibly because of lower rises of temperatures in the computed models.
